# Correlation Between Body Mass Index and Immunotherapy Response in Advanced NSCLC

**DOI:** 10.3390/cancers17071149

**Published:** 2025-03-29

**Authors:** Walid Shalata, Itamar Gothelf, Yulia Dudnik, Ahron Yehonatan Cohen, Ashraf Abu Jama, Tom Liba, Ofir Dan, Lena Tourkey, Sondos Shalata, Abed Agbarya, Amichay Meirovitz, Alexander Yakobson

**Affiliations:** 1 The Legacy Heritage Cancer Center, Dr. Larry Norton Institute, Soroka Medical Center, Beer-Sheva 8410501, Israel; 2 Medical School for International Health, Faculty of Health Sciences, Ben-Gurion University of the Negev, Beer-Sheva 8410501, Israel; 3 Goldman Medical School, Faculty of Health Sciences, Ben-Gurion University of the Negev, Beer-Sheva 8410501, Israel; 4 Azrieli Faculty of Medicine, Bar-Ilan University, Safed 5290002, Israel; 5 Nutrition Unit, Galilee Medical Center, Nahariya 2210006, Israel; 6 Oncology Department, Bnai Zion Medical Center, Haifa 3104701, Israel

**Keywords:** non-small cell lung cancer, body mass index, immune checkpoint inhibitors, overall survival, progression-free survival

## Abstract

Background: Immune checkpoint inhibitors (ICIs) have revolutionized the management of advanced non-small cell lung cancer (NSCLC). Emerging evidence suggests a potential association between elevated body mass index (BMI) and enhanced ICI efficacy, yet this relationship remains inconclusive and warrants further investigation. This study aims to evaluate the impact of BMI on treatment efficacy and survival outcomes in advanced NSCLC patients treated with first-line ICI therapy. Methods: A retrospective study was conducted at a multi-center registry to evaluate the impact of baseline BMI on overall survival (OS) and progression-free survival (PFS) in patients with stage IV NSCLC who received first-line ICI therapies. Treatment regimens included pembrolizumab or the combination of ipilimumab and nivolumab, administered either as monotherapy or in combination with chemotherapy, at the oncology department between January 2018 and December 2023. BMI was categorized according to the World Health Organization (WHO) classification, and OS and PFS were evaluated using Kaplan–Meier survival analysis and the Cox proportional hazards regression model. Results: Among 346 patients, 12.72% were underweight, 45.38% normal weight, 29.19% overweight, and 12.72% obese. Overweight and obese patients were more likely to receive pembrolizumab (*p* = 0.039) and less likely to undergo chemotherapy (*p* = 0.012). No significant differences in median overall survival (OS, log-rank: *p* = 0.155) or progression-free survival (PFS, log-rank: *p* = 0.370) were observed across BMI categories. However, differences emerged upon further analysis of PD-L1 levels (OS, log-rank: *p* = 0.029; PFS, log-rank: *p* = 0.044), additional chemotherapy (OS, log-rank: *p* = 0.009; PFS, log-rank: *p* = 0.021), type of immune checkpoint inhibitor (OS, log-rank: *p* < 0.001; PFS, log-rank: *p* < 0.001), and histologic diagnosis (OS, log-rank: *p* = 0.011; PFS, log-rank: *p* = 0.003). Conclusions: BMI was not an independent predictor of survival outcomes in advanced NSCLC treated with ICI. Incorporating BMI with other patient-specific factors into personalized immunotherapy strategies highlights the importance of tailored approaches to improve patient care and clinical outcomes.

## Simple Summary

An elevated BMI has been proposed to potentially enhance the efficacy of ICIs in advanced NSCLC, although the evidence remains inconsistent, highlighting the need for further research. This study aims to examine the relationship between the BMI of lung cancer patients and their response to immunotherapy. It found no independent link between BMI and survival outcomes in NSCLC patients undergoing first-line ICI treatment. However, PD-L1 expression, chemotherapy use, ICI type, and tumor histology emerged as significant predictors of survival across various BMI categories. These results underscore the necessity for a more tailored approach to immunotherapy, by considering BMI along with other clinical factors to refine treatment strategies and inform future studies on predictive markers.

## 1. Introduction

Immune checkpoint inhibitors (ICIs) have revolutionized cancer treatment, particularly in advanced non-small cell lung cancer (NSCLC), by improving patient survival and achieving durable tumor control [1,2]. These therapies, which target key immune regulatory pathways such as programmed cell death protein 1 (PD-1), its ligand PD-L1, and cytotoxic T-lymphocyte-associated antigen 4 (CTLA-4), represent a significant advancement in the pursuit of long-term disease management and potential remission in select patient populations [3,4]. Despite the significant advancements provided by ICIs, only a limited proportion of patients derive sustained benefits from these treatments. [5]. The majority ultimately experience some form of resistance—whether primary, adaptive, or acquired—that eventually results in disease progression. This resistance is a complex and heterogeneous process, shaped by interactions between the host immune system and the tumor microenvironment, with many underlying mechanisms still not fully understood [6,7].

Several studies have highlighted PD-L1 expression and tumor mutation burden as key biomarkers predicting the effectiveness of PD-1 blockade therapy. [8,9]. Recent findings have pointed to potential host-related predictors of ICI efficacy, including preexisting autoimmune antibodies, C-reactive protein levels, corticosteroid use, white blood cell counts, lactate dehydrogenase levels, and gut microbiota composition [10,11]. Emerging evidence suggests that obesity may play a significant role in modulating patient responses to immunotherapy. Obesity, defined by the World Health Organization (WHO) as a body mass index (BMI) of ≥30 kg/m^2^ [12], is a recognized risk factor for cancer and is frequently linked to poorer outcomes across various malignancies [13]; however, its influence in the context of immunotherapy appears to be more complex. Retrospective studies have indicated a potential association between higher BMI and improved progression-free survival (PFS) and overall survival (OS) in patients treated with ICIs, including those with NSCLC, melanoma, and renal cell carcinoma [14,15].

Elevated BMI has been correlated to improved efficacy in tumor types where PD-1/PD-L1 ICIs demonstrate significant effectiveness, particularly in NSCLC [16]. A study of patients treated with atezolizumab found that obesity conferred a survival advantage compared to normal-weight individuals, an effect not observed in those receiving chemotherapy [17]. Notably, this survival benefit was primarily observed in PD-L1-positive NSCLC patients treated with ICIs, while no significant advantage was detected in PD-L1-negative patients.

This phenomenon may be attributed to the immune-modulating effects of adiposity-induced inflammation. Increased levels of adipokines, such as leptin, in individuals with obesity have been shown to modulate immune function by upregulating pro-inflammatory cytokines and PD-1 expression. These alterations may enhance immune checkpoint activity, thereby augmenting the therapeutic response to ICIs. However, conflicting evidence exists. One study reported no significant difference in PFS or OS between low- and high-BMI groups [18], while another study revealed a non-linear relationship, where mortality risk declined as BMI increased from 20 to 30 kg/m^2^, but began to rise again when BMI exceeded 30 kg/m^2^ [19]. Additionally, studies have shown that high BMI was associated with superior OS in stage IV NSCLC patients treated with chemotherapy, [20] and in early-stage NSCLC patients who underwent lung resections. [21] This suggests that the observed survival benefits of high BMI in NSCLC patients may not be exclusive to immunotherapy but could reflect a broader impact on treatment outcomes across different therapeutic modalities.

Despite growing evidence suggesting an association between elevated BMI and improved efficacy of ICIs, this relationship remains inconclusive and requires further investigation. The present study evaluated the clinical outcomes of two distinct ICI treatment protocols—pembrolizumab versus ipilimumab/nivolumab—while evaluating the impact of BMI on treatment efficacy and survival outcomes in patients with advanced NSCLC. By exploring the interplay between BMI and treatment efficacy, this study aimed to provide novel insights into the role of host-related factors in optimizing immunotherapy outcomes.

## 2. Materials and Methods

### 2.1. Study Population

This retrospective, multi-center registry analyzed a cohort of patients diagnosed with stage IV NSCLC. The treatment regimens included ICI, specifically pembrolizumab or the combination of ipilimumab and nivolumab, administered either as monotherapy or in combination with chemotherapy. All patients received treatment at the oncology centers in Israel between January 2018 and December 2023.

### 2.2. BMI Evaluation

BMI was assessed using patient weight and height data obtained from medical records, either at the initiation of ICI therapy or within four weeks prior to the first dose. BMI was calculated as weight in kilograms divided by height in meters squared (kg/m^2^) and analyzed as a categorical variable according to the World Health Organization (WHO) classifications: underweight (BMI < 18.5), normal weight (18.5–24.9), overweight (25–29.9), and obesity (BMI ≥ 30).

### 2.3. Study Design

This study was designed to evaluate the impact of baseline BMI levels on treatment efficacy and survival outcomes in NSCLC patients receiving ICIs. The primary outcome was OS, measured as the duration from the initial ICI dose to death from any cause. Patients who remained alive were censored at the time of their most recent follow-up. The secondary outcome was PFS, defined as the interval between the first ICI dose and either radiologically confirmed disease progression or death, the earliest of the two events. PFS was assessed based on the Response Evaluation Criteria in Solid Tumors (RECIST), version 1.1. [22] First, we described patients’ characteristics across different BMI groups to establish a clear understanding of the cohort. Univariate analyses were then performed to assess the associations between BMI and the outcome. Based on the univariate findings and supported by the existing literature, we developed a multivariable regression model to evaluate the impact of BMI on treatment efficacy and survival outcomes while accounting for potential confounders. The covariates included in the multivariable analysis were BMI (normal weight, underweight, overweight, obese), histologic type (adenocarcinoma, squamous cell carcinoma [SCC], adeno-squamous carcinoma), gender (female vs. male), age (<70 vs. ≥70 years), smoking status (never, current or past smoker), Eastern Cooperative Oncology Group Performance Status (ECOG-PS; 0–1 vs. ≥2), prior chemotherapy treatment, type of immunotherapy (ipilimumab/nivolumab vs. pembrolizumab), PD-L1 expression levels (<1%, 1–49% and >50%), contra-lateral lung metastasis, lymph node metastasis, brain metastasis, and liver metastasis. For the survival analysis, BMI was further categorized into low BMI (BMI < 25) and high BMI (BMI ≥ 25) to evaluate its impact, along with other patient characteristics, on OS and PFS.

### 2.4. Exclusion Criteria

Exclusion criteria included patients treated with chemotherapy only, cases with unknown BMI values, detection of tumor mutations in EGFR, ALK, ROS, RET, and BRAF, and patients diagnosed with two malignant primaries. These criteria were implemented to ensure the homogeneity and integrity of the study population, facilitating clearer analysis and interpretation of the research findings.

### 2.5. Statistical Analysis

Descriptive statistics were utilized to summarize the baseline demographic, clinical, and molecular characteristics of the patients. Continuous data with non-normal distributions are expressed as medians (range), while categorical variables are presented as frequencies (percentages). Comparisons of patient characteristics across different BMI groups, categorized according to the WHO classification, were performed using either Fisher’s exact test or Pearson’s chi-square test, as appropriate. Kaplan–Meier survival analyses were performed to assess OS and PFS across BMI classifications (low vs. high BMI) with further stratification based on key clinical characteristics, including PD-L1 expression levels, chemotherapy use, type of ICI, gender, histological diagnosis, and age. Log-rank tests were conducted to determine the statistical significance of differences between the survival distributions. Multivariable analyses of PFS and OS were conducted using the Cox proportional hazard regression models to estimate hazard ratios (HRs) along with their corresponding 95% confidence intervals (CIs). All *p*-values were two-sided with a significance level set at *p* < 0.05. The analyses were performed using SPSS software, version 29.0.

## 3. Results

A total of 346 patients participated in this study. Table 1 presents the characteristics of the study population, stratified into subgroups according to the BMI classification. Among the study population, 44 patients (12.72%) were classified as underweight, 157 patients (45.38%) as normal weight, 101 patients (29.19%) as overweight, and 44 patients (12.72%) as obese. The study population had a median age of 67 years (range: 37–87 years), with males comprising the majority (68.5%). A total of 205 patients (59.2%) received pembrolizumab as their ICI, while 141 patients (40.8%) were treated with a combination of ipilimumab and nivolumab, and 285 patients (82.37%) additionally underwent chemotherapy. Regarding PD-L1 expression, 127 patients (36.7%) exhibited levels below 1%, 90 patients (26.0%) had expression ranging from 1 to 49%, and 129 patients (37.3%) showed expression exceeding 50%.

Socio-demographic, diagnostic, and pathological characteristics were compared across different BMI subgroups. Obese and overweight patients were more likely to receive pembrolizumab than the combination of ipilimumab and nivolumab (*p* = 0.039) and were less likely to undergo chemotherapy (*p* = 0.012).

**Figure 1:**
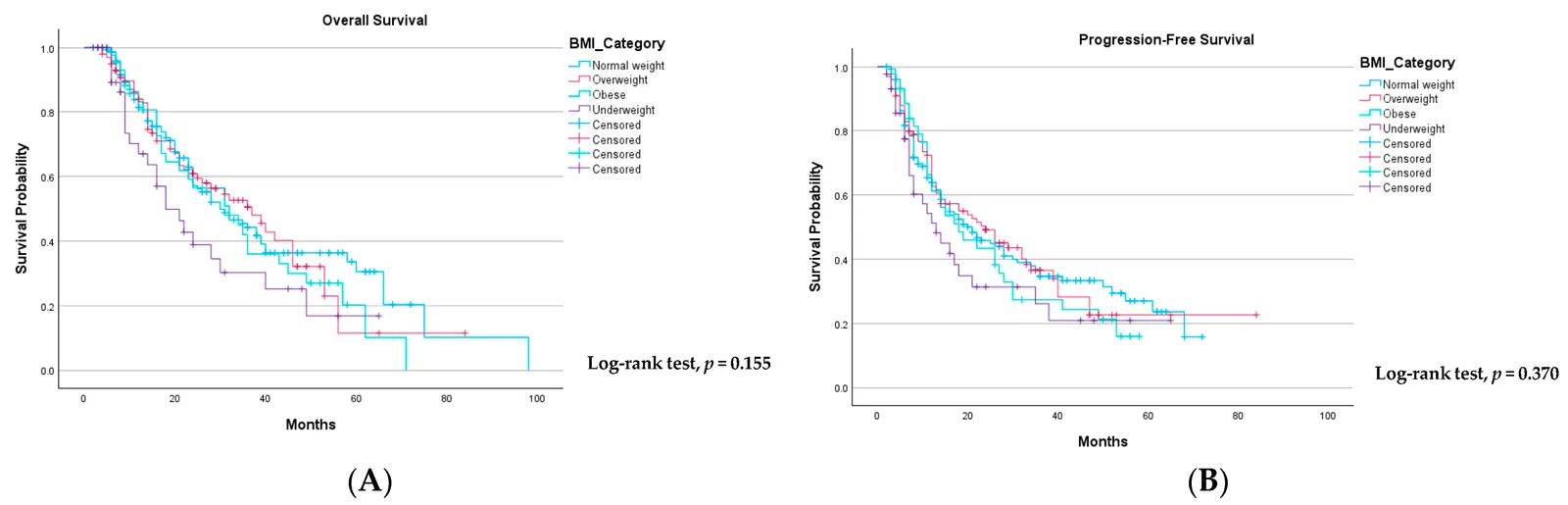
Log-rank test results for overall survival and progression-free survival stratified by BMI categories according to World Health Organization classification. (**A**) Overall Survival: The median overall survival was 18.0 months (95% CI: 10.55 to 25.45) for patients with underweight BMI, 30.0 months (95% CI: 21.47 to 38.53) for those with normal weight, 37.0 months (95% CI: 26.38 to 47.62) for overweight patients, and 32.0 months (95% CI: 19.84 to 44.16) for obese patients. The log-rank test indicated no statistically significant difference in survival between the BMI groups (*p* = 0.155). (**B**) Progression-Free Survival: The median progression-free survival was 13.0 months (95% CI: 6.61 to 19.39) for patients with underweight BMI, 21.0 months (95% CI: 14.17 to 27.83) for those with normal weight, 24.0 months (95% CI: 14.20 to 33.80) for overweight patients, and 18.0 months (95% CI: 8.30 to 27.70) for obese patients. The log-rank test showed no significant difference between the BMI groups (*p* = 0.370).

Figure 1 presents Kaplan–Meier survival analyses for the study population, illustrating OS (Figure 1A) and PFS (Figure 1B) stratified by BMI classification (low vs. high). The log-rank test indicated no statistically significant differences in OS (*p* = 0.155) or PFS (*p* = 0.370) between BMI categories.

**Figure 2:**
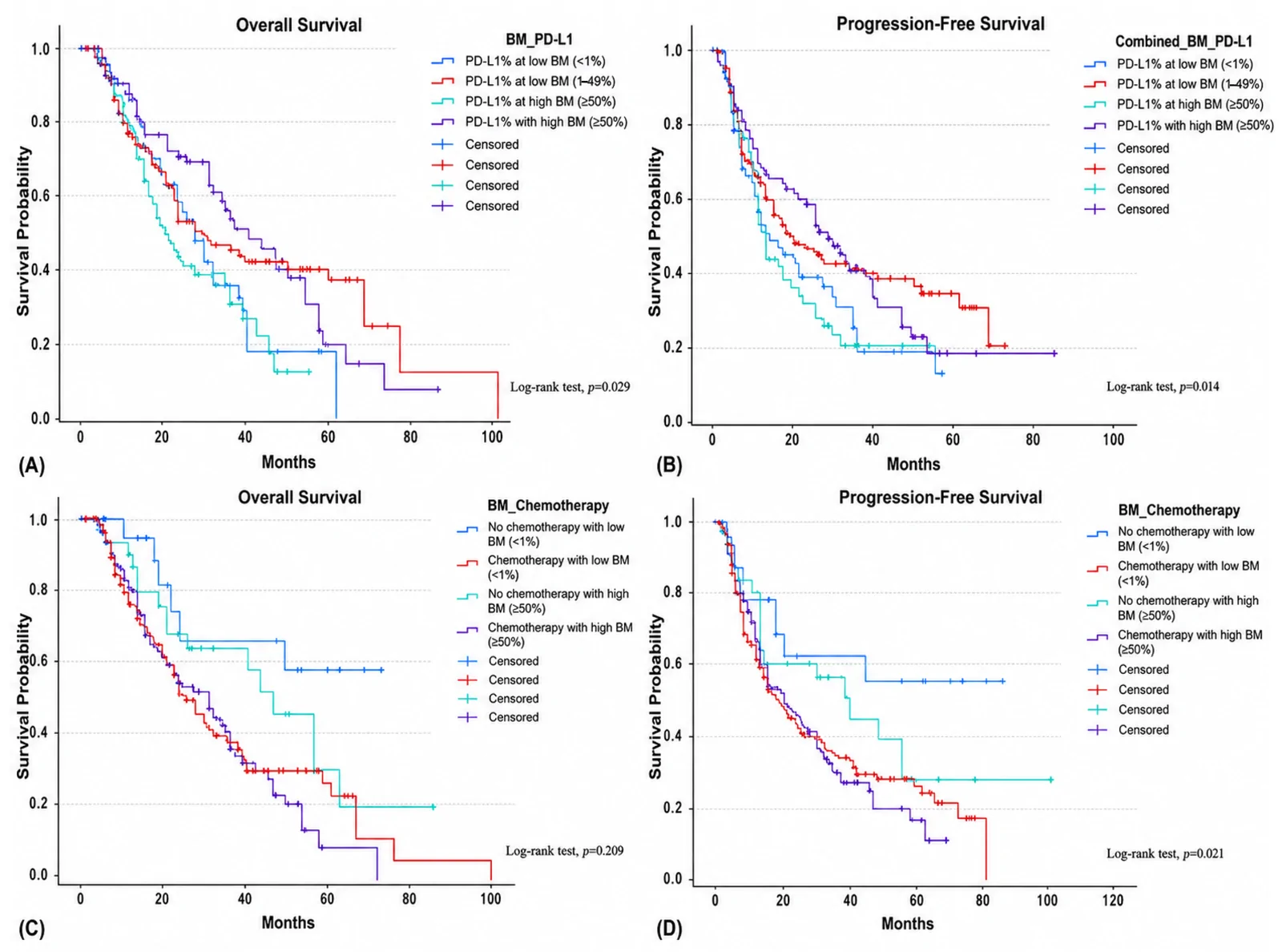
Log-rank test results for overall survival and progression-free survival stratified by BMI in combination with PD-L1 levels and chemotherapy status. (**A**) Overall Survival: The median overall survival was 28.0 months (95% CI: 22.44 to 33.56) for patients with PDL1 < 1% and low BMI (*n* = 66), 30.0 months (95% CI: 19.61 to 40.39) for patients with PDL1 ≥ 1% and low BMI (*n* = 139), 21.0 months (95% CI: 15.30 to 26.70) for patients with PDL1 < 1% and high BMI (*n* = 61), and 40.0 months (95% CI: 29.96 to 50.03) for patients with PDL1 ≥ 1% and high BMI (*n* = 79). The log-rank test indicated a statistically significant survival difference between the groups (*p* = 0.029). (**B**) Progression-Free Survival: The median progression-free survival was 15.0 months (95% CI: 8.24 to 21.76) for PDL1 < 1% and low BMI, 20.0 months (95% CI: 10.82 to 29.18) for PDL1 ≥ 1% and low BMI, 14.0 months (95% CI: 12.35 to 15.64) for PDL1 < 1% and high BMI, and 29.0 months (95% CI: 21.48 to 36.52) for PDL1 ≥ 1% and high BMI. The log-rank test showed a statistically significant difference between the groups (*p* = 0.044). (**C**) Overall Survival: The median overall survival was not estimable for patients with no chemotherapy and low BMI (*n* = 26), 28.0 months (95% CI: 23.48 to 32.52) for patients with chemotherapy and low BMI (*n* = 177), 46.0 months (95% CI: 33.47 to 58.53) for patients with no chemotherapy and high BMI (*n* = 31), and 31.0 months (95% CI: 21.85 to 40.15) for patients with chemotherapy and high BMI (*n* = 106). The log-rank test indicated a statistically significant survival difference between the groups (*p* = 0.009). (**D**) Progression-Free Survival: The median progression-free survival was not estimable for patients with no chemotherapy and low BMI, 17.0 months (95% CI: 12.35 to 21.65) for patients with chemotherapy and low BMI, 34.0 months (95% CI: 16.31 to 51.69) for patients with no chemotherapy and high BMI, and 18.0 months (95% CI: 12.41 to 23.59) for patients with chemotherapy and high BMI. The log-rank test showed a statistically significant difference between the groups (*p* = 0.021).

Figure 2 presents Kaplan–Meier survival analyses for OS and PFS stratified by BMI categories (low vs. high) in combination with key clinical factors. OS and PFS were first analyzed by BMI categories and PD-L1 levels (Figure 2A and Figure 2B, respectively), showing significant differences (log-rank, *p* = 0.029 and *p* = 0.044, respectively). Additionally, OS (Figure 2C) and PFS (Figure 2D) were examined by BMI categories and chemotherapy use (log-rank, *p* = 0.009 and *p* = 0.021, respectively).

**Figure 3:**
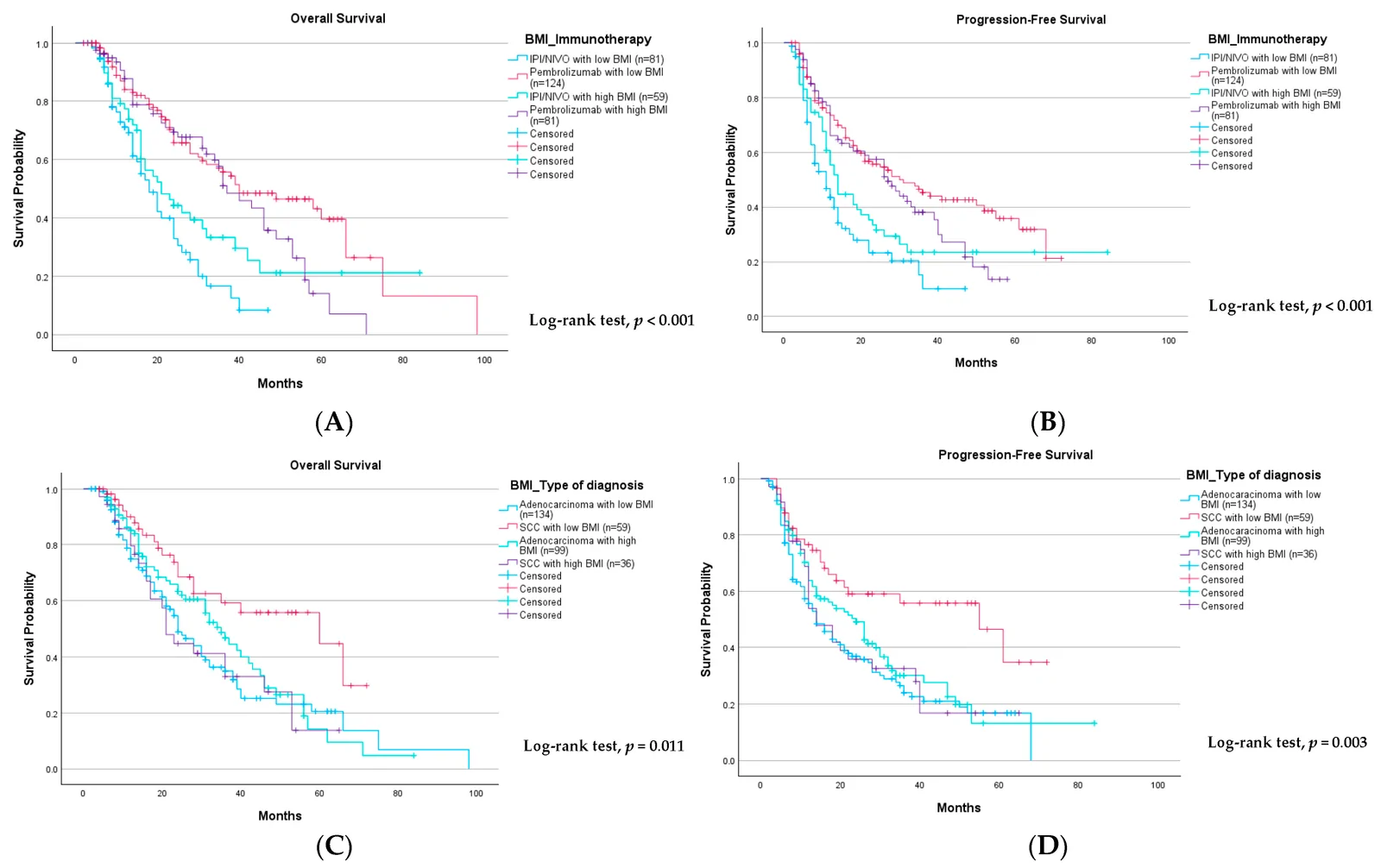
Log-rank test results for overall survival and progression-free survival stratified by BMI (low vs. high) in combination with type of immunotherapy and histologic diagnosis. (**A**) Overall Survival: The median overall survival was 18.0 months (95% CI: 13.90 to 22.10) for patients treated with IPI/NIVO and low BMI (*n* = 81), 40.0 months (95% CI: 21.30 to 58.70) for those treated with pembrolizumab and low BMI (*n* = 124), 21.0 months (95% CI: 13.20 to 28.80) for those treated with IPI/NIVO and high BMI (*n* = 59), and 37.0 months (95% CI: 29.50 to 44.50) for those treated with pembrolizumab and high BMI (*n* = 81). The log-rank test indicated a statistically significant survival difference between the groups (*p* < 0.001). (**B**) Progression-Free Survival: The median progression-free survival was 11.0 months (95% CI: 7.26 to 14.74) for patients treated with IPI/NIVO and low BMI, 30.0 months (95% CI: 17.67 to 42.33) for those treated with pembrolizumab and low BMI, 14.0 months (95% CI: 11.60 to 16.40) for those treated with IPI/NIVO and high BMI, and 27.0 months (95% CI: 20.00 to 33.96) for those treated with pembrolizumab and high BMI. The log-rank test showed a statistically significant difference between the groups (*p* < 0.001). (**C**) Overall Survival: The median overall survival was 24.0 months (95% CI: 19.38 to 28.62) for patients with adenocarcinoma and low BMI (*n* = 134), 60.0 months (95% CI: 18.09 to 101.91) for those with SCC and low BMI (*n* = 59), 35.0 months (95% CI: 28.80 to 41.20) for those with adenocarcinoma and high BMI (*n* = 99), and 21.0 months (95% CI: 16.93 to 25.07) for those with SCC and high BMI (*n* = 36). The log-rank test indicated a statistically significant survival difference between the groups (*p* = 0.011). (**D**) Progression-Free Survival: The median progression-free survival was 14.0 months (95% CI: 9.77 to 18.23) for patients with adenocarcinoma and low BMI, 55.0 months (95% CI: 29.49 to 80.51) for those with SCC and low BMI, 24.0 months (95% CI: 17.17 to 30.83) for those with adenocarcinoma and high BMI, and 14.0 months (95% CI: 9.64 to 18.36) for those with SCC and high BMI. The log-rank test showed a statistically significant difference between the groups (*p* = 0.003).

Figure 3 presents an analysis of overall survival (OS) and progression-free survival (PFS) stratified by BMI classification (low vs. high) in conjunction with key clinical characteristics. Stratification by BMI and immunotherapy regimen (Figure 3A,B) revealed statistically significant differences in OS or PFS (log-rank, *p* < 0.001 for both). Similarly, stratification by BMI and histologic subtype (Figure 3C,D) demonstrated significant differences in OS (*p* = 0.011) and PFS (*p* = 0.003). Further Kaplan–Meier analyses stratified by BMI classification and gender (Supplementary Figure S1) demonstrated no statistically significant differences in OS (*p* = 0.868) nor PFS (*p* = 0.576), and a stratification by BMI and age (<70 vs. ≥70 years) (Supplementary Figure S2) showed a trend toward significance for OS (*p* = 0.088), while no significant difference was observed for PFS (*p* = 0.337).

The multivariable Cox regression analysis presented in Table 2 evaluated the impact of BMI on OS and PFS while accounting for potential confounders. No association was observed between BMI categories and treatment efficacy or survival outcomes. The findings indicate that poorer ECOG performance status (2+) was significantly associated with worse PFS (HR = 1.77, *p* = 0.002, 95% CI: 1.24–2.54) and OS (HR = 1.80, *p* = 0.004, 95% CI: 1.21–2.68). Similarly, administration of additional chemotherapy was linked to reduced PFS (HR = 1.81, *p* = 0.013; 95% CI: 1.14–2.88) and OS (HR = 2.23, *p* = 0.002; 95% CI: 1.33–3.75), while the presence of liver metastasis was associated with worse PFS (HR = 1.95, *p* < 0.001; 95% CI: 1.32–2.88) and OS (HR = 1.99, *p* = 0.002; 95% CI: 1.28–3.08). Patients with SCC demonstrated poorer PFS compared to those with adenocarcinoma (HR = 1.57, *p* = 0.042, 95% CI: 1.02–2.43). Treatment with pembrolizumab was associated with improved OS compared to combination therapy with ipilimumab and nivolumab (HR = 0.64, *p* = 0.022; 95% CI: 0.43–0.94), and demonstrated a trend toward enhanced PFS (HR = 0.72, *p* = 0.066; 95% CI: 0.51–1.02). Conversely, the presence of contra-lateral lung metastases was linked to worse OS (HR = 1.45, *p* = 0.027; 95% CI: 1.04–2.01) and showed a similar trend in PFS (HR = 1.28, *p* = 0.10; 95% CI: 0.95–1.71).

## 4. Discussion

This study aimed to evaluate the association between BMI and the therapeutic efficacy of ICIs and to identify key factors modulating this relationship. The study comprised several key components: (a) characterization of the patient population, stratified by BMI subgroups; and (b) evaluation of OS and PFS across the BMI subgroups, along with an analysis of the influence of additional patient characteristics on these associations. In this study, no significant association was observed between BMI and clinical outcomes in patients with NSCLC treated with first-line ICIs. However, BMI was found to be associated with clinical outcomes when analyzed in the context of different PD-L1 levels, additional chemotherapy treatment, type of ICI, and histologic diagnosis.

Emerging evidence suggests that obese patients may experience improved outcomes with ICIs [17,22]. Obesity introduces a complex interplay between inflammation and immune dysfunction. Chronic low-grade systemic inflammation associated with obesity disrupts normal immune homeostasis, impairing the function of T cells. The inflammatory milieu in obesity contributes to T cell exhaustion, marked by an upregulation of PD-1-positive dysfunctional T cells with diminished tumor-fighting capacity. This state of exhaustion is likely influenced by immune checkpoint pathways, such as PD-1/PD-L1, and further amplified by leptin, a hormone frequently elevated in obesity [23]. Paradoxically, these same factors may increase the responsiveness of obese patients to immune checkpoint blockade therapies, highlighting unique therapeutic opportunities within this population [24]. Furthermore, recent research suggests a potential role for white adipose tissue in immune regulation. Preclinical studies in mice have demonstrated that this tissue can serve as a reservoir for pathogen-specific memory T cells following pathogenic infections [25]. These memory T cells, including tissue-resident subsets characterized by distinct metabolic properties, may facilitate a rapid immune response upon reactivation. These findings raise the possibility that adipose tissue-resident T cells could similarly be reactivated to target cancer-specific antigens.

On the other hand, preclinical studies have demonstrated that obesity may significantly alter the tumor microenvironment, creating conditions that promote tumor growth and metastasis [26]. One mechanism involves an increased presence of tumor-infiltrating dendritic cells. In the context of obesity, these dendritic cells adopt an immunosuppressive phenotype, leading to the inhibition of CD8+ T cell activity and thereby impairing the immune system’s ability to mount an effective antitumor response [27].

In our study, while the previously suggested association between elevated BMI and improved outcomes in patients receiving first-line ICI therapy did not reach statistical significance, we found that overweight patients achieved the most favorable OS and PFS. These results are consistent with the findings of Cortellini et al., who also observed no significant correlation between elevated BMI and clinical outcomes but noted that overweight patients demonstrated the most prolonged OS [28]. The treatment regimen in our study population may explain the absence of statistical significance. While some studies have demonstrated improved OS and PFS with immunotherapy alone, this benefit has not been consistently observed when combined with chemotherapy [14,16,17]. As the majority of patients in our cohort received the combined therapy, this treatment approach may have influenced our findings. Furthermore, variations in BMI classification thresholds across studies may contribute to discrepancies in reported outcomes, highlighting the need for standardized BMI cut-off points in future research. Notably, while some studies found no significant difference in OS or PFS when BMI was analyzed as a binary variable (high vs. low BMI), they did observe significant differences when patients were stratified into four BMI categories [16,18]

Interestingly, when OS and PFS were stratified by BMI categories and PD-L1 expression levels (<50% vs. ≥50%), the subgroup of overweight patients with high PD-L1 expression (≥50%) exhibited the most favorable outcomes. This finding may reflect a synergistic effect in this subgroup, combining obesity-related enhancements in immune responsiveness, as previously described, with the well-established association between high PD-L1 expression and improved efficacy of ICIs [29].

Furthermore, stratification by BMI categories and the use of additional chemotherapy revealed a significant difference in OS among the groups. Overweight and obese patients who received ICI-based therapy without chemotherapy demonstrated the longest OS. The addition of chemotherapy to ICIs may attenuate the obesity-related enhancement in immune responsiveness by broadly suppressing immune cell populations and altering the tumor microenvironment. A study by Cortellini et al. similarly evaluated survival and disease progression outcomes among patients who received first-line ICIs and a control group treated with first-line chemotherapy [30]. The study found that obesity was associated with longer OS in the ICI group, whereas no such association was observed in the chemotherapy group. These findings further highlight the more pronounced correlation between BMI and outcomes in patients treated with ICIs compared to those receiving chemotherapy.

To the best of our knowledge, this is the first study to evaluate the effect of different immunotherapies on survival and disease progression outcomes across BMI categories in patients with advanced NSCLC. We identified differences in OS and PFS associated with the type of ICI administered. Overweight and obese patients demonstrated improved OS and PFS when treated with pembrolizumab compared to ipilimumab/nivolumab. Unlike pembrolizumab, a PD-1 inhibitor that specifically targets the obesity-driven PD-1/PD-L1 pathway, ipilimumab, a CTLA-4 inhibitor, modulates the immune response through a broader mechanism of action [31]. This broader immunomodulation may be less directly influenced by the enhanced immune responsiveness associated with obesity, potentially accounting for the differing therapeutic outcomes observed between these two approaches.

This study has several limitations. The retrospective nature of the analysis may introduce inherent biases, making it challenging to determine causal relationships between variables. As a result, the findings are limited to identifying associations rather than establishing definitive causation. Additionally, our study did not include data on comorbidities, particularly those commonly associated with obesity, such as hypertension, diabetes mellitus, dyslipidemia, and other cardiovascular diseases. The reliance on BMI as a proxy for obesity has inherent limitations, as it does not differentiate between adipose tissue and skeletal muscle mass, nor does it provide information on the distribution of adipose tissue, such as subcutaneous vs. visceral fat [32]. Future research incorporating detailed body composition analysis is essential to better understand the impact of adiposity on cancer-related outcomes. Furthermore, a multiple dynamic evaluation of BMI changes throughout treatment would provide a more comprehensive understanding of its immunological effects compared to relying solely on a single baseline or pretreatment BMI measurement. As a real-world study, our findings reflect variability in treatment administration and patient monitoring compared to controlled trials. Unlike the standardized protocols of clinical trials, real-world practice varies across institutions in imaging frequency, response assessment, and ECOG evaluations, introducing heterogeneity in recorded outcomes. This underscores the need for rigorous documentation and standardized real-world data collection to improve comparability and reliability in observational research. Finally, the prognostic influence of BMI has been found to vary among different cancer types, indicating that the applicability of our findings may be limited to NSCLC and may not extend to other tumor types.

## 5. Conclusions

This study provides important insights into the influence of BMI on survival and disease progression outcomes in advanced NSCLC patients treated with first-line ICI therapy. While no direct association was identified between BMI and clinical outcomes, stratification by additional patient characteristics revealed notable correlations. The type of ICI treatment, histological features, and use of additional therapies were found to be influenced by BMI categories, ultimately impacting survival and disease progression outcomes. Future research should focus on prospective study designs, dynamic assessments of BMI throughout treatment, and advanced body composition analyses to enhance our understanding of the relationship between adiposity and outcomes in cancer immunotherapy. These findings emphasize the critical role of incorporating BMI and other patient-specific factors into clinical decision-making and the development of personalized immunotherapy strategies to optimize treatment outcomes in advanced NSCLC.

## Figures and Tables

**Table 1:** Baseline characteristics of the study population stratified by BMI categories according to WHO classification.

Characteristics	Overall (%)	Underweight (%)	Normal Weight (%)	Overweight (%)	Obese (%)	*p*-Value
	*N* = 346	*N* = 44	*N* = 157	*N* = 101	*N* = 44	
Age (years)						
Median (IQR)	67.0 (62.0; 74.0)	64.5 (58.3; 74.8)	67.0 (62.0; 74.0)	69.0 (64.0; 75.0)	65.0 (60.0; 73.0)	0.114
Range	37–87	38–86	37–83	39–87	47–82	
<70	199 (57.51)	28 (63.64)	90 (57.32)	54 (53.47)	27 (61.36)	
>70	147 (42.49)	16 (36.36)	67 (42.68)	47 (46.53)	17 (38.64)	
Gender, *N* (%)						
Female	109 (31.5)	19 (43.18)	49 (31.2)	26 (25.7)	15 (34.1)	0.214
Male	237 (68.5)	25 (56.8)	108 (68.8)	75 (74.3)	29 (65.9)	
Histology, *N* (%)						
Adenocarcinoma	233 (67.3)	28 (63.6)	106 (67.5)	70 (69.3)	29 (65.9)	0.383
Squamous cell	96 (27.7)	12 (27.3)	43 (27.4)	29 (28.7)	12 (27.3)	
carcinoma						
Adenosquamous	8 (2.3)	2 (4.5)	3 (1.9)	0 (0)	3 (6.8)	
Other	9 (2.6)	2 (4.5)	5 (3.2)	2 (2.0)	0 (0)	
Smoking status, *N* (%)						
Never	45 (13.0)	6 (13.6)	17 (10.9)	15 (14.9)	7 (15.9)	0.438
Current	174 (50.4)	24 (54.5)	84 (53.8)	51 (50.5)	15 (34.1)	
Past	124 (35.9)	14 (31.8)	54 (34.6)	35 (34.7)	21 (47.7)	
Unknown	2 (0.6)	0 (0)	1 (0.6)	0 (0)	1 (2.3	
ECOG, *N* (%)						
0	72 (20.9)	9 (20.5)	34 (21.7)	20 (20.0)	9 (20.5)	0.869
1	200 (58.0)	23 (52.3)	89 (56.7)	63 (63.0)	25 (56.8)	
2+	73 (21.2)	12 (27.3)	34 (21.7)	17 (17.0)	10 (22.7)	
Type of Immunotherapy, *N* (%)						
Pembrolizumab	205 (59.2)	18 (40.9)	102 (65.0)	60 (59.4)	25 (56.8)	0.039
Ipilimumab and	141 (40.8)	26 (59.1)	55 (35.0)	41 (40.6)	19 (43.2)	
Nivolumab						
Chemotherapy, *N* (%)						
No	58 (16.76)	4 (9.3)	20 (12.8)	27 (27.0)	7 (15.9)	0.012
Yes	285 (82.37)	39 (90.7)	136 (87.2)	73 (73.0)	37 (84.1)	
Unknown	3 (0.86)					
Type of chemotherapy, *N* (%)						
Carboplatin-based	269 (94.7)	38 (97.4)	128 (94.8)	68 (93.2)	35 (94.6)	0.816
Cisplatin-based	15 (5.3)	1 (2.6)	7 (5.2)	5 (6.8)	2 (5.4)	
Contra-lateral lung metastasis, *N* (%)						
No	173 (50.0)	21 (47.7)	86 (54.8)	46 (45.5)	20 (45.5)	0.442
Yes	173 (50.0)	23 (52.3)	71 (45.2)	55 (54.5)	24 (54.5)	
Lymph node metastasis, *N* (%)						
No	147 (42.5)	21 (47.7)	65 (41.4)	42 (41.6)	19 (43.2)	0.894
Yes	199 (57.5)	23 (52.3)	92 (58.6)	59 (58.4)	25 (56.8)	
Pleural metastasis, *N* (%)						
No	281 (81.2)	35 (79.5)	125 (79.6)	81 (80.2)	40 (90.9)	0.373
Yes	65 (18.8)	9 (20.5)	32 (20.4)	20 (19.8)	4 (9.1)	
Pericardial metastasis, *N* (%)						
No	337 (97.4)	44 (100.0)	155 (98.7)	96 (95.0)	42 (95.5)	0.163
Yes	9 (2.6)	0 (0)	2 (1.3)	5 (5.0)	2 (4.5)	
Brain metastasis, *N* (%)						
No	285 (82.4)	37 (84.1)	130 (82.8)	84 (83.2)	34 (77.3)	0.815
Yes	61 (17.6)	7 (15.9)	27 (17.2)	17 (16.8)	10 (22.7)	
Bone metastasis, *N* (%)						
No	240 (69.4)	29 (65.9)	118 (75.2)	64 (63.4)	29 (65.9)	0.196
Yes	106 (30.6)	15 (34.1)	39 (24.8)	37 (36.6)	15 (34.1)	
Adrenal metastasis, *N* (%)						
No	299 (86.4)	38 (86.4)	133 (84.7)	91 (90.1)	37 (84.1)	0.624
Yes	47 (13.6)	6 (13.6)	24 (15.3)	10 (9.9)	7 (15.9)	
Liver metastasis, *N* (%)						
No	303 (87.6)	38 (86.4)	137 (87.3)	89 (88.1)	39 (88.6)	0.986
Yes	43 (12.4)	6 (13.6)	20 (12.7)	12 (11.9)	5 (11.4)	
Spleen metastasis, *N* (%)						
No	343 (99.1)	44 (100.0)	155 (98.7)	100 (99.0)	44 (100.0)	0.780
Yes	3 (0.9)	0 (0)	2 (1.3)	1 (1.0)	0 (0)	
PD-L1 expression, *N* (%)						
<1%	127 (36.7)	12 (27.3)	54 (34.4)	39 (38.6)	22 (50.0)	0.315
1–49%	90 (26.0)	16 (36.4)	42 (26.8)	24 (23.8)	8 (18.2)	
>50%	129 (37.3)	16 (36.4)	61 (38.9)	38 (37.6)	14 (31.8)	
Tumor mutational burden						
Median (range)	7.0 (0.95–75)	5.90 (0.95–27.16)	8.61 (1.0–41.10)	6.60 (0.95–75.0)	7.85 (1.0–19.0)	0.132
Unknow	127 (36.7)	11 (25.0)	57 (36.3)	41 (40.6)	18 (40.9)	
FGFR molecular status, *N* (%)						
Wild type	334 (96.5)	42 (95.5)	151 (96.2)	97 (96.0)	44 (100.0)	0.601
Mutant	12 (3.5)	2 (4.5)	6 (3.8)	4 (4.0)	0 (0)	
KRAS molecular status, *N* (%)						
Wild type	281 (81.2)	31 (70.5)	132 (84.1)	81 (80.2)	37 (84.1)	0.213
Mutant	65 (18.8)	13 (29.5)	25 (15.9)	20 (19.8)	7 (15.9)	
STK-11 molecular status, *N* (%)						
Wild type	322 (93.1)	42 (95.5)	141 (89.8)	97 (96.0)	42 (95.5)	0.192
Mutant	24 (6.9)	2 (4.5)	16 (10.2)	4 (4.0)	2 (4.5)	
TP-53 molecular status, *N* (%)						
Wild type	268 (77.5)	38 (86.4)	121 (77.1)	74 (73.3)	35 (79.5)	0.371
Mutant	78 (22.5)	6 (13.6)	36 (22.9)	27 (26.7)	9 (20.5)	

**Table 2:** Multivariable Cox regression analyses of patient characteristics and their association with progression-free survival and overall survival.

Variable	Adjusted HR for PFS; CI 95%	*p*-Value for PFS	Adjusted HR for OS; CI 95%	*p*-Value for OS
Body mass index				
Normal weight	1		1	
Underweight	0.89; 0.65–1.22	0.467	0.90; 0.65–1.24	0.515
Overweight	0.94; 0.75–1.18 0.86	0.587	0.87; 0.68–1.12	0.280
Obese	0.67–1.11	0.239	0.86; 0.65–1.13	0.265
Histology				
Adenocarcinoma	1		1	
Squamous cell	1.57; 1.02–2.43	0.042	1.23; 0.79–1.92	0.353
carcinoma				
Adeno-squamous	1.19; 0.74–1.90	0.474	1.11; 0.69–1.80	0.651
Other	0.95; 0.38–2.38	0.916	1.17; 0.46–2.94	0.747
Gender				
Female	1		1	
Male	1.25; 0.90–1.73	0.181	1.20; 0.84–1.71	0.308
Age				
<70	1		1	
>70	0.91; 0.68–1.22	0.517	1.06; 0.77–1.47	0.718
Smoking status				
Never	1			
Current	1.23; 0.92–1.64	0.161	1.30; 0.95–1.79	0.107
Past	0.98; 0.80–1.21	0.874	0.96; 0.76–1.20	0.704
ECOG				
0–1	1		1	
2+	1.77; 1.24–2.54	0.002	1.80; 1.21–2.68	0.004
Chemotherapy				
No	1		1	
Yes	1.81; 1.14–2.88	0.013	2.23; 1.33–3.75	0.002
Type of Immunotherapy				
Ipilimumab and	1		1	
Nivolumab				
Pembrolizumab	0.72; 0.51–1.02	0.066	0.64; 0.43–0.94	0.022
PD-L1 expression				
<1%	1		1	
1–49%	1.17; 0.94–1.45	0.160	1.09; 0.85–1.39	0.510
>50%	0.97; 0.78–1.20	0.777	0.99; 0.78–1.25	0.921
Contra-lateral lung metastasis				
No	1		1	
Yes	1.28; 0.95–1.71	0.10	1.45; 1.04–2.01	0.027
Lymph node metastasis				
No	1		1	
Yes	0.76; 0.57–1.02	0.071	0.78; 0.56–1.08	0.135
Brain metastasis				
No	1		1	
Yes	0.99; 0.68–1.43	0.951	0.98; 0.65–1.48	0.927
Liver metastasis				
No	1		1	
Yes	1.95; 1.32–2.88	<0.001	1.99; 1.28–3.08	0.002

## Data Availability

The data either reside within the article itself or can be obtained from the authors upon making a reasonable request.

## Supplementary Materials

The following supporting information can be downloaded at: https://www.mdpi.com/article/10.3390/cancers17071149/s1, Figure S1. Log-rank test results for overall survival and progression-free survival stratified by BMI (low vs. high) and gender. Figure S2. Log-rank test results for overall survival and progression-free survival stratified by BMI (low vs. high) and age (<70 vs ≥70) years.
