# Correction: Shalata et al. Correlation Between Body Mass Index and Immunotherapy Response in Advanced NSCLC. *Cancers* 2025, *17*, 1149

**DOI:** 10.3390/cancers18172791

**Published:** 2026-08-28

**Authors:** Walid Shalata, Itamar Gothelf, Yulia Dudnik, Ahron Yehonatan Cohen, Ashraf Abu Jama, Tom Liba, Ofir Dan, Lena Tourkey, Sondos Shalata, Abed Agbarya, Amichay Meirovitz, Alexander Yakobson

**Affiliations:** 1 The Legacy Heritage Cancer Center, Dr. Larry Norton Institute, Soroka Medical Center, Beer-Sheva 8410501, Israel; 2 Medical School for International Health, Faculty of Health Sciences, Ben-Gurion University of the Negev, Beer-Sheva 8410501, Israel; 3 Goldman Medical School, Faculty of Health Sciences, Ben-Gurion University of the Negev, Beer-Sheva 8410501, Israel; 4 Azrieli Faculty of Medicine, Bar-Ilan University, Safed 5290002, Israel; 5 Nutrition Unit, Galilee Medical Center, Nahariya 2210006, Israel; 6 Oncology Department, Bnai Zion Medical Center, Haifa 3104701, Israel

## Error in Figure

In the original publication [1], there was a mistake in Figure 2 as published. There has been a change to Figure 2B, instead of less (<), it has been changed to more (>) for the description of PDL1 status and low BMI (*n* = 139), this was to correct the mistake of: PDL1 < 1% and low BMI (*n* = 139), instead of PDL1 > 1% and low BMI (*n* = 139). The corrected Figure 2 appears below. The authors state that the scientific conclusions are unaffected. This correction was approved by the Academic Editor. The original publication has also been updated.

## Figures and Tables

**Figure 2:**
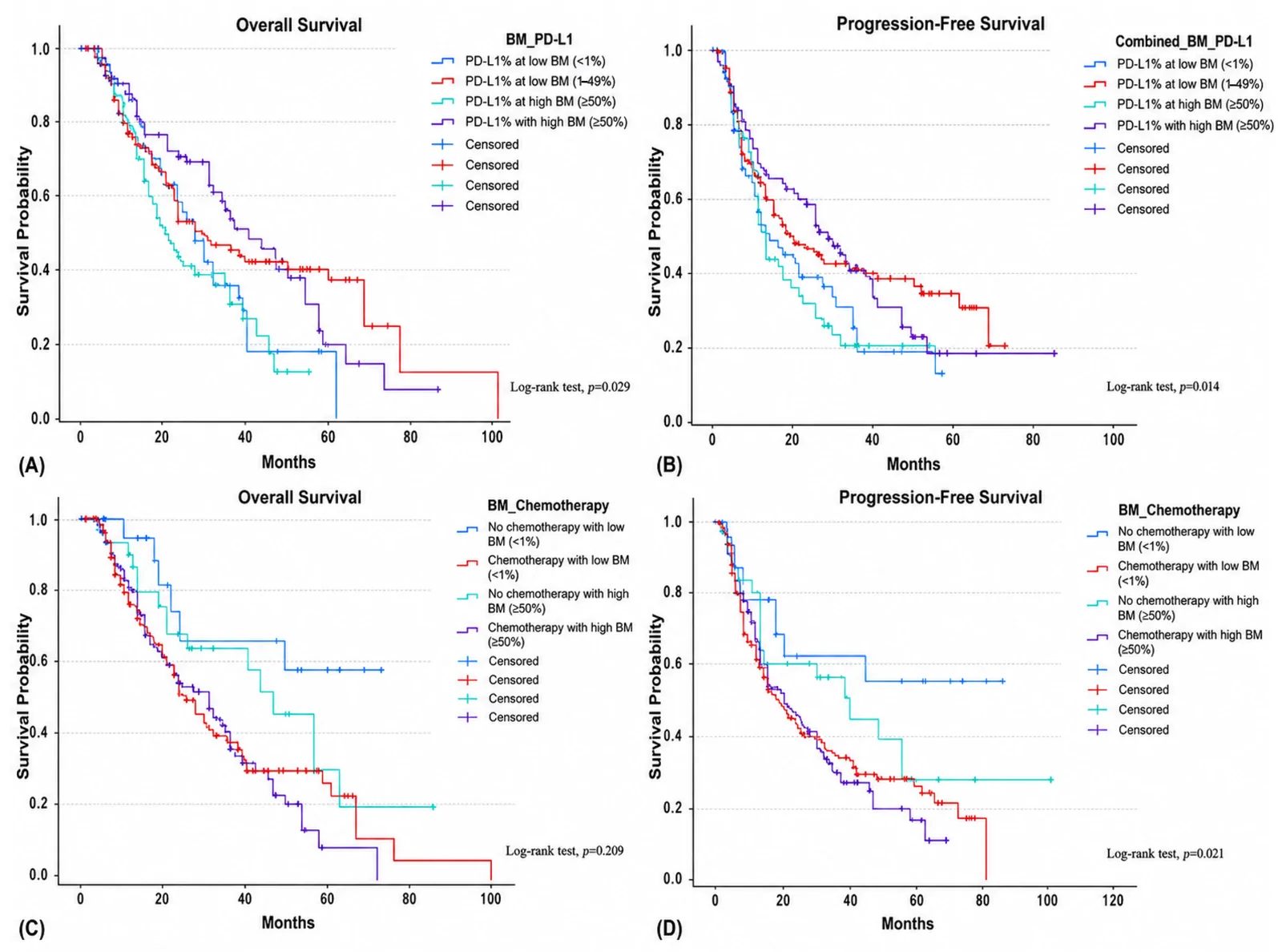
Log-rank test results for overall survival and progression-free survival stratified by BMI in combination with PD-L1 levels and chemotherapy status. (**A**) Overall Survival: The median overall survival was 28.0 months (95% CI: 22.44 to 33.56) for patients with PDL1 < 1% and low BMI (*n* = 66), 30.0 months (95% CI: 19.61 to 40.39) for patients with PDL1 ≥ 1% and low BMI (*n* = 139), 21.0 months (95% CI: 15.30 to 26.70) for patients with PDL1 < 1% and high BMI (*n* = 61), and 40.0 months (95% CI: 29.96 to 50.03) for patients with PDL1 ≥ 1% and high BMI (*n* = 79). The log-rank test indicated a statistically significant survival difference between the groups (*p* = 0.029). (**B**) Progression-Free Survival: The median progression-free survival was 15.0 months (95% CI: 8.24 to 21.76) for PDL1 < 1% and low BMI, 20.0 months (95% CI: 10.82 to 29.18) for PDL1 ≥ 1% and low BMI, 14.0 months (95% CI: 12.35 to 15.64) for PDL1 < 1% and high BMI, and 29.0 months (95% CI: 21.48 to 36.52) for PDL1 ≥ 1% and high BMI. The log-rank test showed a statistically significant difference between the groups (*p* = 0.044). (**C**) Overall Survival: The median overall survival was not estimable for patients with no chemotherapy and low BMI (*n* = 26), 28.0 months (95% CI: 23.48 to 32.52) for patients with chemotherapy and low BMI (*n* = 177), 46.0 months (95% CI: 33.47 to 58.53) for patients with no chemotherapy and high BMI (*n* = 31), and 31.0 months (95% CI: 21.85 to 40.15) for patients with chemotherapy and high BMI (*n* = 106). The log-rank test indicated a statistically significant survival difference between the groups (*p* = 0.009). (**D**) Progression-Free Survival: The median progression-free survival was not estimable for patients with no chemotherapy and low BMI, 17.0 months (95% CI: 12.35 to 21.65) for patients with chemotherapy and low BMI, 34.0 months (95% CI: 16.31 to 51.69) for patients with no chemotherapy and high BMI, and 18.0 months (95% CI: 12.41 to 23.59) for patients with chemotherapy and high BMI. The log-rank test showed a statistically significant difference between the groups (*p* = 0.021).
