# Supplementary material for: Correlation Between Body Mass Index and Immunotherapy Response in Advanced NSCLC
Source: Cancers (Basel). 2025 Mar 29;17(7):1149. doi: 10.3390/cancers17071149 (PMC11988004; doi:10.3390/cancers17071149)
Supplement: Supplementary file 1 [file cancers-17-01149-s001.zip › cancers-3527849-supplementary.pdf]

Figure S1. Log-rank test results for overall survival and progression-free survival stratified by BMI (low vs. high) and gender.

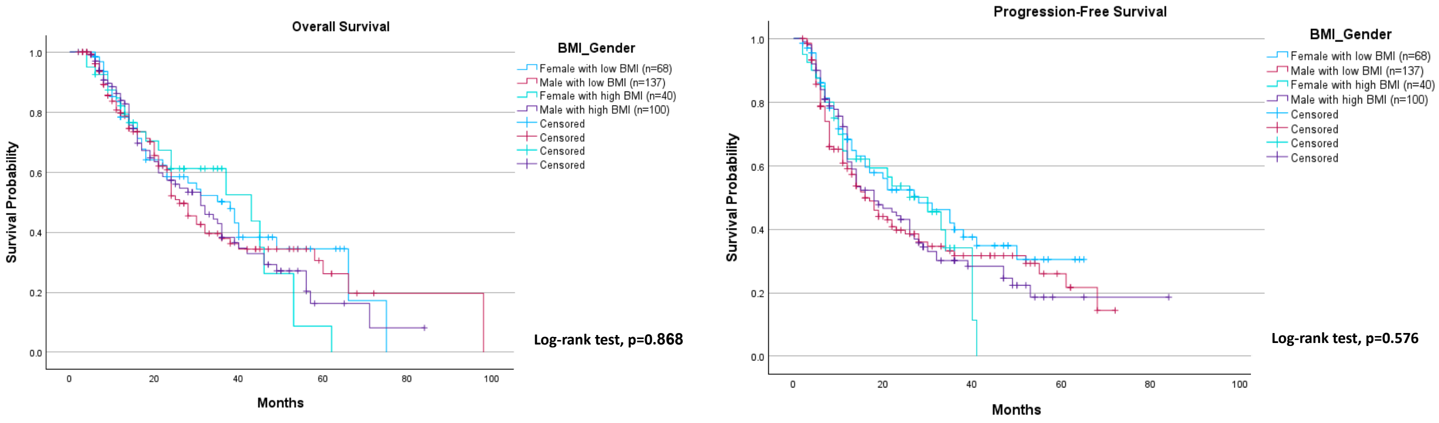

(A) **(B)**

(A) Overall Survival: The median overall survival was 38.0 months (95% CI: 26.9 to 49.1) for females with low BMI (n=68), 26.0 months (95% CI: 22.0 to 30.0) for males with low BMI (n=137), 43.0 months (95% CI: 17.4 to 68.5) for females with high BMI (n=40), and 31.0 months (95% CI: 22.5 to 39.5) for males with high BMI (n=100). The log-rank test indicated no statistically significant difference in survival between the groups (p = 0.868). (B) Progression-Free Survival: The median progression-free survival was 28.0 months (95% CI: 14.2 to 41.8) for females with low BMI, 16.0 months (95% CI: 11.9 to 20.1) for males with low BMI, 30.0 months (95% CI: 17.7 to 42.3) for females with high BMI, and 18.0 months (95% CI: 11.1 to 24.9) for males with high BMI. The log-rank test showed no significant difference between the groups (p = 0.576).

Figure S2. Log-rank test results for overall survival and progression-free survival stratified by BMI (low vs. high) and age (<70 vs ≥70) years.

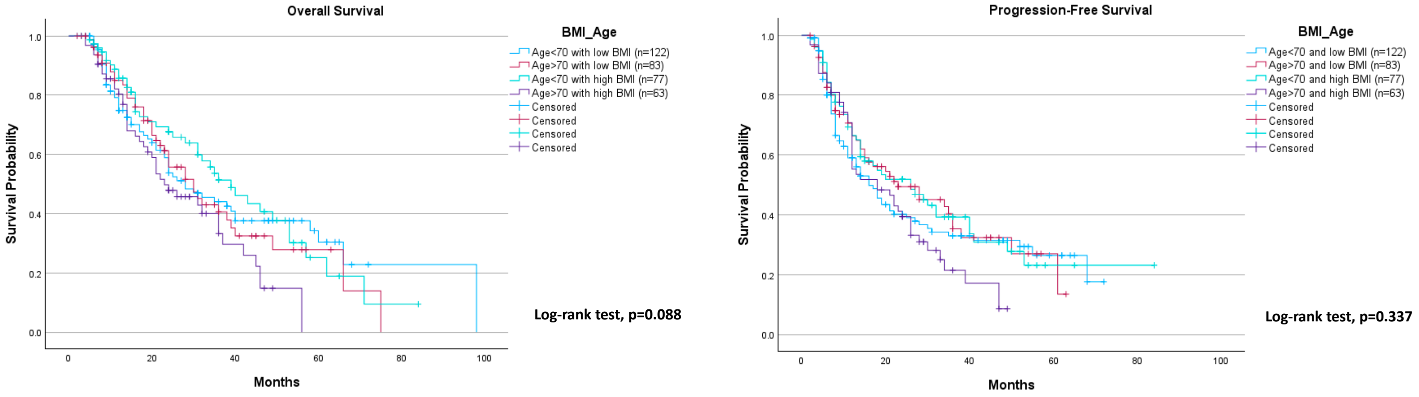

(A) **(B)**

(A) Overall Survival: The median overall survival was 28.0 months (95% CI: 19.3 to 36.7) for patients under 70 years with low BMI (n=122), 30.0 months (95% CI: 21.9 to 38.1) for those over 70 years with low BMI (n=83), 39.0 months (95% CI: 29.2 to 48.8) for patients under 70 years with high BMI (n=77), and 23.0 months (95% CI: 14.1 to 31.9) for those over 70 years with high BMI (n=63). The log-rank test indicated no statistically significant difference in survival between the groups (p = 0.088). (B) Progression-Free Survival: The median progression-free survival was 16.0 months (95% CI: 11.5 to 20.5) for patients under 70 years with low BMI, 23.0 months (95% CI: 8.1 to 37.9) for those over 70 years with low BMI, 26.0 months (95% CI: 14.5 to 37.5) for patients under 70 years with high BMI, and 18.0 months (95% CI: 7.6 to 28.4) for those over 70 years with high BMI. The log-rank test showed no significant difference between the groups (p = 0.337).
